# Bio-inspired TiO_2_ nano-cone antireflection layer for the optical performance improvement of VO_2_ thermochromic smart windows

**DOI:** 10.1038/s41598-020-68411-6

**Published:** 2020-07-09

**Authors:** Sai Liu, Chi Yan Tso, Hau Him Lee, Yi Zhang, Kin Man Yu, Christopher Y. H. Chao

**Affiliations:** 1 School of Energy and Environment, City University of Hong Kong, Tat Chee Avenue, Kowloon, Hong Kong China; 2 Department of Mechanical Engineering, The University of Hong Kong, Hong Kong, China; 3 Department of Physics, City University of Hong Kong, Tat Chee Avenue, Kowloon, Hong Kong China

**Keywords:** Energy science and technology, Applied optics

## Abstract

Vanadium dioxide (VO_2_) is a promising material for thermochromic glazing. However, VO_2_ thermochromic smart windows suffer from several problems that prevent commercialization: low luminous transmittance (*T*_*lum*_) and low solar modulation ability (*ΔT*_*sol*_). The solution to these problems can be sought from nature where the evolution of various species has enabled them to survive. Investigations into the morphology of moths eyes has shown that their unique nanostructures provide an excellent antireflection optical layer that helps moths sharply capture the light in each wavelength from a wide angle. Inspired by this mechanism, a VO_2_ thermochromic smart window coated with a TiO_2_ antireflection layer with a novel nano-cone structure, is presented in this study to achieve high *T*_*lum*_ and *ΔT*_*sol*_. Optimization for the key structure parameters is summarized based on the FDTD numerical simulations. The optimized structure exhibits a *T*_*lum*_ of 55.4% with *ΔT*_*sol*_ of 11.3%, an improvement of about 39% and 72% respectively compared to the VO_2_ window without an antireflection layer. Furthermore, wide-angle antireflection and polarization independence are also demonstrated by this nano-cone coating. This work provides an alternative method to enhance the optical performance of VO_2_ smart windows.

## Introduction

Energy and environmental problems have become critical issues in modern society. It has been proven that buildings consume 20–40% of the total energy used. Most importantly, 60% of that energy is consumed to maintain thermal comfort by heating, ventilation and air conditioning (HVAC) systems^1^. The huge consumption of energy by HVAC systems is mainly due to heat loss through the building envelope such as the roof, walls and windows. Among these building envelopes, approximately 50% of the energy is lost through windows, so more attention has been focused on energy efficient windows^2^. Thermochromic smart windows are widely investigated because of their low cost and passive controllability of solar irradiance^3^. Vanadium dioxide (VO_2_) is one of the thermochromic materials to modulate transmittance of solar radiation because of its internal reversible phase change from metal (hot state) to insulator state (cold state) at a critical transition temperature of 68 °C^4^. The phase change gives rise to an abrupt change of the near-infrared (NIR) transmittance, which renders VO_2_ a promising candidate for thermochromic smart windows. To fulfill the demand of human vision and energy efficiency, improving luminous transmittance (*T*_*lum*_) and solar modulation ability (i.e. the difference of solar transmittance (*T*_*sol*_) between cold and hot states of materials or *ΔT*_*sol*_) simultaneously are key for the practical applications of VO_2_ smart windows in buildings^5^. Many efforts have been made to improve *T*_*lum*_ and *ΔT*_*sol*_ of thermochromic smart windows, such as chemical doping^6,7^, synthesizing VO_2_ nanoparticles^8–11^ and morphology modification^12–15^. Among the different methods, depositing a planar antireflection layer is a commonly used solution to achieve high *T*_*lum*_ due to the simple fabrication process^16,17^ and high reflectance of VO_2_^18^ . The principle of the antireflection coating can be explained by destructive interference of two reflected light beams from air–coating and coating–substrate interfaces, thereby cancelling each other and increasing the transmittance. For a destructive interference to occur, the optical thickness of the film (*h*) must be an odd multiple of *λ/4*, where *λ* is the wavelength of the incident light^19^, and *h* can be calculated as Eq. (1),

1 $$ h = nd, $$

where *n* is the refractive index of the material and *d* is the thickness of the layers. For a double-layer film structure with two dielectric films, there are normally two commonly used planar antireflection optical structures as shown in Supplementary Fig. S1. One is a quarter-quarter-waved structure (Supplementary Fig. S1a), and the other is a quarter-three quarter-waved structure (Supplementary Fig. S1b). The quarter-quarter-waved structure contains two films which have equal *h* as the quarter wavelength (*λ/4*). For the quarter-three quarter-waved structures, one film has the quarter-waved optical thickness (*h* = *λ/4*), the other film has the three quarter-waved optical thickness (*h* = *3λ/4*).

Apart from the optical thickness of the antireflection layers, the refractive index of each layer also plays an important role to influence the antireflection performance. Based on the Fresnel equation, normal-incidence reflection can be minimized if the refractive index of the top layer can fulfill the condition^19^:

2 $$ n_{1} = n_{2} \sqrt {\frac{{n_{0} }}{{n_{s} }}} , $$

where, *n*_*s*_ = refractive index of the substrate, *n*_*0*_ = refractive index of air, *n*_*1*_ = refractive index of the top layer, *n*_*2*_ = refractive index of the bottom layer.

By carefully selecting a suitable material (refractive index) and thickness of antireflection layer, a desired state of antireflection can be achieved. Based on these guidelines, researchers apply various materials as planar antireflection layers to improve the optical performance of VO_2_ smart windows. TiO_2_ is one of the most widely used materials due to its matching refractive index with VO_2_^16,20^. P. Jin’s group first designed a double-layer-antireflection coating with TiO_2_/VO_2_ and reported increases of the *T*_*lum*_ from 32 to 49% and the *ΔT*_*sol*_ from 4.4 to 7.0%^21^. Later, a three layered TiO_2_/VO_2_/TiO_2_ structure was proposed by the same group^22^ achieving a maximum increase in *T*_*lum*_ of 86% (i.e. from 30.9 to 57.6%) but with an undesirable drop of *ΔT*_*sol*_ from 3.9 to 2.9%^23^. Furthermore, N. R. Mlyuka’s group proposed a TiO_2_/VO_2_/TiO_2_/VO_2_/TiO_2_ five layered structure, and *T*_*lum*_ increased from 40.5 to 43.65% while *ΔT*_*sol*_ increased from 6.7 to 12.1% compared with the VO_2_ single layer^24^. Although previous reports using antireflective coatings demonstrated some improvements in the *T*_*lum*_ or *ΔT*_*sol*_, significant simultaneous improvements in the *T*_*lum*_ and *ΔT*_*sol*_ of VO_2_ remain challenging. This is because the continuous thin film antireflection layer can only reduce the reflection at certain wavelengths, and it is hard to achieve a broadband wavelength (300 nm‒2.5 µm in this study) antireflection performance^25^. While the target wavelength to improve *T*_*lum*_ is in the visible light region, the thermochromic effect (*∆T*_*sol*_) occurs primarily in the NIR (> 800 nm) region. Hence, it is difficult to design a planar antireflection structure to achieve the reflectance in both the visible light region and NIR region. Alternatively, nanostructure arrays are proposed as the antireflective coating to achieve antireflection in both the visible and NIR region owing to their refractive index gradient^25^.

**Figure 1:**
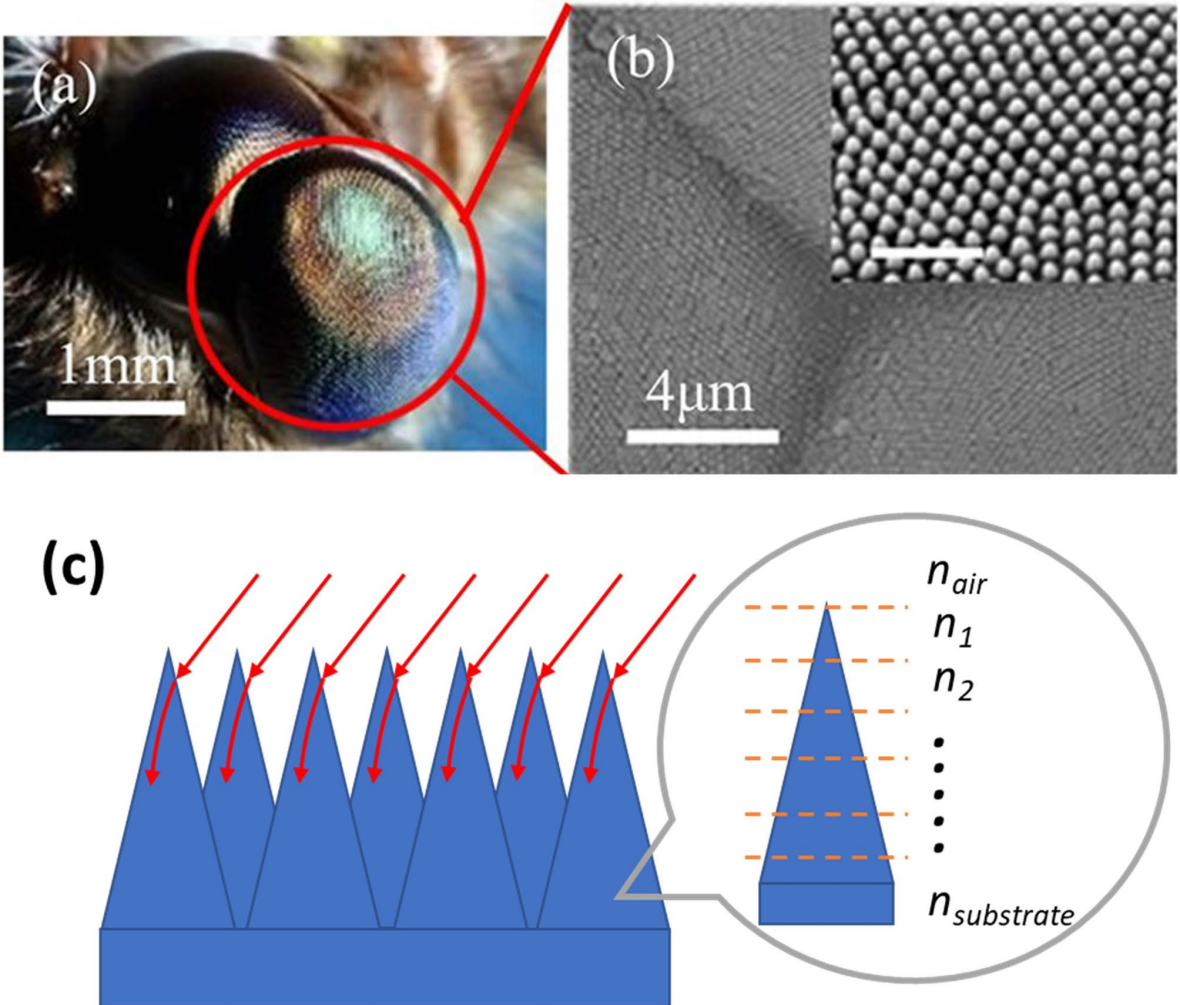
(**a**) The moth eye and (**b**) its SEM image showing the nano-structure (Figures by J.Y. SUN et al*.*^35^ are licensed under CC BY 4.0) (**c**) Interaction of incident light with moth eye structure.

Inspired by moth eyes^26–28^, researchers investigated the 3D nano-cone antireflection structure and found that it can dramatically suppress reflection and improve light transmission^29^. Most importantly, this 3D nano-cone structure provides antireflection ability in broadband wavelength and it is insensitive to the direction and polarization of the incident light^27,28^, which helps moths capture the light in each wavelength from a wide angle (Fig. 1a). The nano-cone surface is composed of tapered arrays with dimensions less than the incident light wavelength. The nanoscale array provides a gradual refractive index change from the top of the cone where the refractive index is *n*_*air*_ = 1 to the bottom with a higher refractive index. Because of the refractive index gradient, the incident light is insensitive to the structures and tends to bend progressively into the material (Fig. 1b,c)^19,25^. Although the angle of incidence changes, the coating still exhibits a relatively smooth change of refractive index, thereby suppressing the reflection of incidence in a broad range of wavelength. Furthermore, the super-hydrophobic property of the nanoscale array promotes the self-cleaning function which is helpful to address contamination problems on the antireflection surface^30,31^. Therefore, this kind of nanoscale coating has the perfect properties, namely, high transmittance in the broadband wavelength, polarization-insensitivity and self-cleaning. There are some reports of using a nano-cone antireflection layer on solar cells to reduce the light reflection and achieve good optical and energy harvesting performance^32–34^.

In 2013, Taylor et al. designed nanostructured, densely packed SiO_2_ paraboloidal protrusions coated with a single thin layer of VO_2_ on a smart window. Based on their Finite-difference Time-domain (FDTD) simulation results, the *ΔT*_*sol*_ can reach 23.1% while simultaneously maintaining a high *T*_*lum*_ of 70.3%^23^. The optimized results were achieved when the width of the cone was about 130 nm with height greater than 500 nm, and the coated VO_2_ thin film was less than 10 nm. However, the large aspect ratio and the small thickness of the VO_2_ layer pose great difficulties in fabrication of the structure. Following the simulation by Taylor et al., Qian et al. fabricated nanostructure VO_2_ smart windows (with a periodicity of 440 nm and VO_2_ film thickness of 140 nm), and reported the *T*_*lum*_ of only 44.5%, with *ΔT*_*sol*_ of 7.1%^5^.

This work aims to achieve high *T*_*lum*_ and *ΔT*_*sol*_ simultaneously for VO_2_ thermochromic smart windows. A novel VO_2_–TiO_2_ nano-cone structure antireflection layer for the thermochromic smart window is proposed since TiO_2_ is an easily obtained and refractive matching material for the antireflection layer. Also, TiO_2_ is frequently employed to treat pollutants and withstand fogging due to its photocatalytic and photo-induced hydrophilic properties, which can help the windows achieve self-cleaning functions^36^. In addition, the refractive index gradient nano-structure can improve the antireflection performance in the broadband wavelength and imprinting techniques have been widely used on TiO_2_ to imprint nano-patterns^37–40^. In order to achieve the best results of *T*_*lum*_ and *ΔT*_*sol*_, FDTD simulations are conducted to examine the best geometrical dimension for the thickness of VO_2_ and TiO_2_ as well as the height and pitch of the nano-cone array. The optimized structure shows that the nano-cone structure offers an improvement of *T*_*lum*_ without deteriorating *ΔT*_*sol*_. Meanwhile, the independence of the polarization and wide-angle of the incident light is demonstrated. This opens a new approach to enhance optical properties for thermochromic VO_2_ smart windows in real-life applications, providing high transmittance along with low energy consumption for buildings.

## Methodology

### Calculation of optical parameters

There are two important indices to quantify the optical performance of the VO_2_ thermochromic smart windows, which are luminous transmittance (*T*_*lum*_) and solar modulation ability (*∆T*_*sol*_).

*T*_*lum*_ describes the amount of visible light transmitted by the windows that is useful for human vision under normal conditions, it is defined in Eq. (3),

3 $$ T_{lum} = \frac{{\mathop \int \nolimits_{\lambda = 380nm}^{780nm} \overline{y}\left( \lambda \right)T\left( \lambda \right)d\lambda }}{{\mathop \int \nolimits_{\lambda = 380nm}^{780nm} \overline{y}\left( \lambda \right)d\lambda }}, $$

where *T(λ)* is the transmittance of the windows at wavelength *λ*. *y ®(λ)* is the CIE (International Commission on Illumination) standard for photopic luminous efficiency of the human eyes. The wavelength range used for *T*_*lum*_ is 380 nm–780 nm, corresponding to the limits of human vision. For convenience in this study, the average luminous transmittance (*T*_*lum,ave*_) which describes the average value of luminous transmittance in the cold (*T*_*lum,cold*_) and hot state (*T*_*lum,hot*_) is defined as,

4 $$ T_{lum,ave} = \frac{{T_{lum,cold} + T_{lum.hot} }}{2}, $$

and hereafter, for simplicity, the symbol *T*_*lum*_ will be used to represent *T*_*lum,ave*_ . Accounting for the NIR entering buildings through windows, the transmittance of NIR (*T*_*IR*_*)* is stated as

5 $$ T_{IR} = \frac{{\mathop \int \nolimits_{\lambda = 780nm}^{2500nm} AM_{1.5} \left( \lambda \right)T\left( \lambda \right)d\lambda }}{{\mathop \int \nolimits_{\lambda = 780nm}^{2500nm} AM_{1.5} \left( \lambda \right)d\lambda }}, $$

where *AM*_*1.5*_* (λ)* is the solar irradiance spectrum for an air mass of 1.5. The AM1.5 weighting spectrum is chosen for *T*_*sol*_ calculations as it represents an overall annual average for mid-latitudes including diffuse light from the ground and sky on a south facing surface tilted 37° from horizontal^41^. The wavelength range for calculation is from 300 to 2,500 nm which accounts for higher than 99% of terrestrial solar energy. Apart from transmittance, the luminous reflectance (*R*_*lum*_)^42^ and absorption (*A*_*lum*_) are calculated by integrating with photopic luminous efficiency as Eqs. (6) and (7),

6 $$ R_{lum} = \frac{{\mathop \int \nolimits_{\lambda = 380nm}^{780nm} \overline{y}\left( \lambda \right)R\left( \lambda \right)d\lambda }}{{\mathop \int \nolimits_{\lambda = 380nm}^{780nm} \overline{y}\left( \lambda \right)d\lambda }}, $$

7 $$ A_{lum} = 1 - T_{lum} - R_{lum} , $$

where *R(λ)* is the reflectance.

In order to quantify the amount of solar thermal energy entering the house through windows, solar transmittance (*T*_*sol*_) is defined as Eq. (8)

8 $$ T_{sol} = \frac{{\mathop \int \nolimits_{\lambda = 300nm}^{2500nm} AM_{1.5} \left( \lambda \right)T\left( \lambda \right)d\lambda }}{{\mathop \int \nolimits_{\lambda = 300nm}^{2500nm} AM_{1.5} \left( \lambda \right)d\lambda }}, $$

and the solar modulation effect of a smart window between cold and hot state is quantified as

9 $$ {\Delta }T_{sol} = T_{sol,cold} - T_{sol,hot} . $$

### Optimization criteria

In a preliminary design of the structure, in order to select the antireflection material with suitable refractive index and predict the thickness of the antireflection layer, one specific wavelength is chosen as the target wavelength for simplicity. Considering the highest photopic luminous efficiency and solar irradiance (green and orange regions, respectively, in Supplementary Fig. S2)^23^, the wavelength of 550 nm is designated as the target wavelength and the main purpose of the antireflection coating is to minimize the reflectance at 550 nm. It should be pointed out that the wavelength of 550 nm is only used to carry out the preliminary design to determine the suitable material as the antireflection material. In the detailed FDTD simulation, the simulated wavelength region is indeed from 300–2,500 nm which covers the whole visible light and solar irradiation range. The wavelength dependent complex refractive index of VO_2_ and TiO_2_ are also considered in the wavelength range of 300–2,500 nm.

### Optimization design

The approximate refractive index (neglecting the extinction coefficient) of VO_2_ at 550 nm is 2.79 in the cold state and 2.44 in the hot state^23^. The result calculated from Eq. (2) shows that the refractive index of the suitable antireflection material should be about 2.30 in the cold state and 2.03 in the hot state. The refractive index of TiO_2_ (2.44 at 550 nm) is closer to that ideal value, justifying our choice of TiO_2_ as the optical antireflection material in this work.

**Figure 2:**
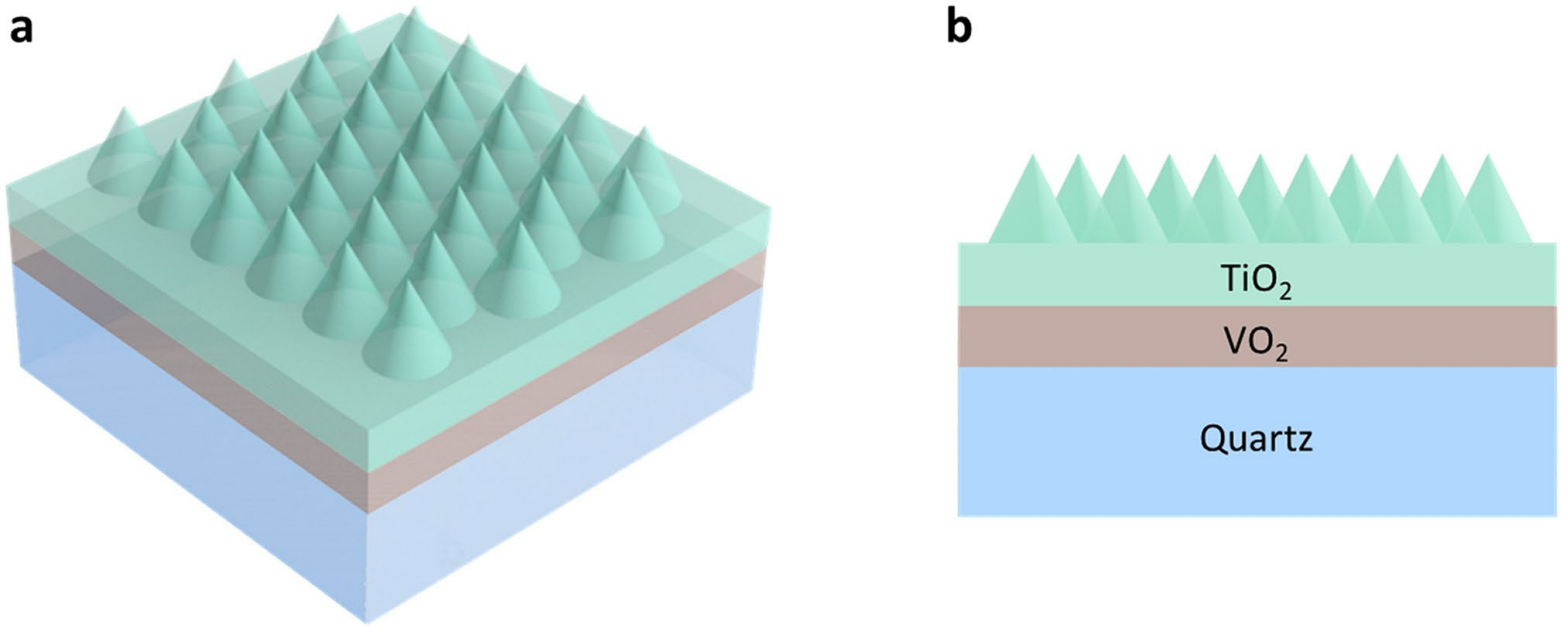
(**a**) 3D schematic diagram and (**b**) cross section of the nano-cone antireflection coating.

Nano-cone antireflection surface was first discovered on the cornea of night-flying moths in 1967^43^. The eyes of this insect are covered with nipples at a pitch ranging from 180 to 240 nm and heights varying between 0 and 230 nm. Their hexagonal arrangement is due to the high areal densities to provide a large area for antireflection^26^. So only a hexagonal nano-cone surface distribution is investigated in this study. To support the nano-cones, a planar TiO_2_ layer is designed between the VO_2_ and TiO_2_ nano-cones. The whole structure is deposited on the quartz substrate in sequence and is schematically illustrated in Fig. 2. For the best optical performance, the thickness of VO_2_ and TiO_2_ planar layers, as well as the pitch (the distance between two adjacent nano-cones) and the height of the TiO_2_ nano-cones are the key parameters to be optimized in the simulation. It should be noted that the main purpose of this study is to investigate the improvement of optical performance by adding nano-cones, so only one planar TiO_2_ antireflection layer is adopted even though multiple layers may lead to better *T*_*lum*_ and *ΔT*_*sol*_*.* The optimization is conducted by a cycling method to ensure the optimized structure can be selected. The detailed optimization steps can be found in Supplementary Fig. S3 and Supplementary Note S1.

### FDTD simulation model

As FDTD gives an excellent scaling performance of the method as the problem size grows and is widely used in the research of nanophononics^33,44–46^, FDTD simulations are conducted to optimize the thickness of each layer as well as the dimensions of the nano-cones and to analyze the optical performance. The real and imaginary parts of the complex refractive indices from 300–2,500 nm of the VO_2_ and TiO_2_ are taken from the references^23,47^. The structure is drawn in the commercial software Lumerical FDTD Solutions as shown in Supplementary Fig. S4. For the structure optimization to determine the suitable thickness of VO_2_ and TiO_2_ as well as the dimensions of the nano-cones, the plane wave propagating along the *Z* direction is used to simulate the normal incident light. Two frequency-dependent monitors are put into the SiO_2_ substrate and on the top of the plane wave source respectively to collect spectral data of transmittance and reflectance. The Perfectly Matched Layer (PML) boundary conditions which absorb propagating and evanescent light waves with minimal reflections are set for the *Z* direction. Symmetric boundary conditions (an equal electromagnetic field through the middle of the simulation region) are applied for the *X* and *Y* directions to reduce the memory requirement. Regarding the investigation of optical performance under different polarized light, Broadband Fixed Angle Source Technique (BFAST) plane wave is used along *X* or *Y* direction to simulate p- or s-polarized light, and the incident angle is set from 0° to 60°.

Validation of the FDTD model is carried out using two different approaches to ensure the accuracy and reliability of our proposed simulation model. Firstly, the *T*_*lum*_ and *∆T*_*sol*_ of the simulation results based on only one-layer of VO_2_ are compared with the previous simulation results in different thicknesses. The comparison as shown in Supplementary Fig. S5 demonstrates that the FDTD simulation results match those previously reported by others^23^. The other validation is conducted to compare with the experimental results^48^, using a glass/TiO_2_/VO_2_/TiO_2_/VO_2_/TiO_2_ five layer structure with 40 nm VO_2_ films and 80 nm TiO_2_ films. The same structure is repeated in our FDTD model for validation. The results show that *T*_*lum*_ = 29.1%, *T*_*sol*_ = 29.7% in the hot state, and *T*_*lum*_ = 35.2%, *T*_*sol*_ = 43.4% in the cold state, which are almost identical to the experimental results of Nuru R. Mlyuka’s study^48^ as shown in Supplementary Table S1. The consistency of these validations proves that the simulation model in this study is accurate and reliable.

## Results and discussion

This study starts the investigation based on normal incidence. The transmittance data from the wavelength of 300–2,500 nm are collected and analyzed to evaluate the optical performance of the smart window. To explore the *T*_*lum*_ and *∆T*_*sol*_ for the TiO_2_ nano-cone antireflection layer, studies have been systematically conducted by varying the four parameters, the thicknesses of VO_2_ and TiO_2_ as well as the pitch and height of the nano-cone, and the results are discussed. Finally, to prove polarization-insensitive anti-reflectivity of the nano-cone structure, the simulations of incidence angles up to 60° under p- and s-polarized light are conducted, and the results are demonstrated.

### The effect of VO_2_ layer thickness on the *T*_lum_ and ∆*T*_sol_

**Figure 3:**
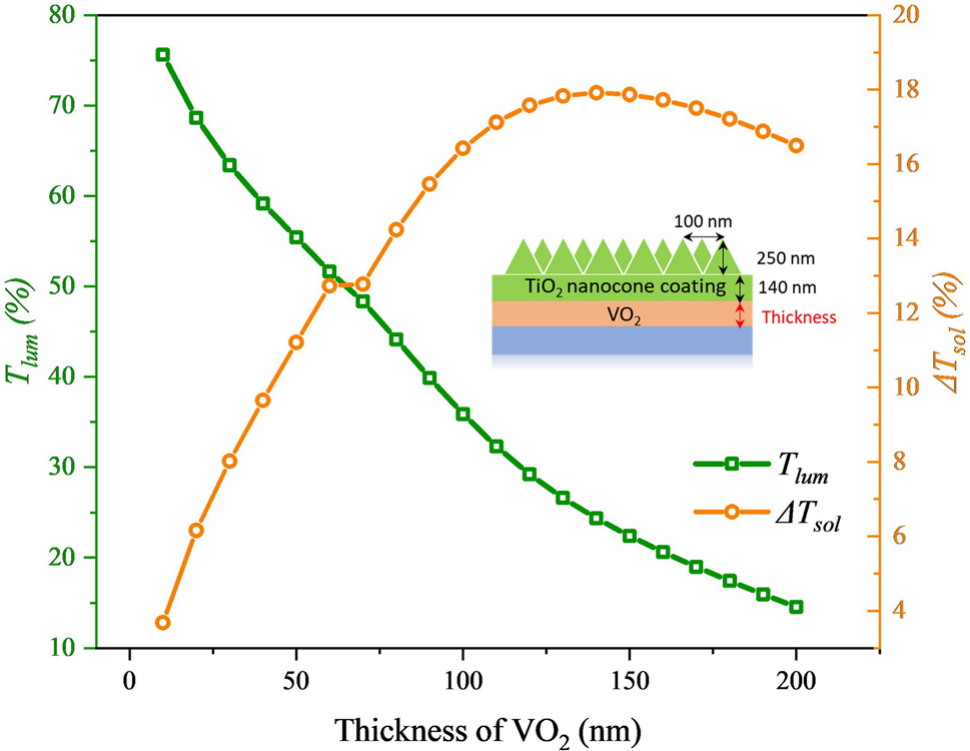
Simulated *T*_*lum*_ and *ΔT*_*sol*_ of different thicknesses of VO_2_.

The thickness of VO_2_ is important to the *T*_*lum*_ and *∆T*_*sol*_. The simulation results shown in Fig. 3 demonstrate the trade-off between the *T*_*lum*_ and *∆T*_*sol*_ with the change of VO_2_ thickness (i.e. 10–200 nm)_,_ while the other parameters are constant (TiO_2_ thickness: 140 nm, pitch: 100 nm, height: 250 nm). As the VO_2_-thickness increases, *∆T*_*sol*_ grew while *T*_*lum*_ decreases. For window applications, the higher *T*_*lum*_ (e.g. > 55%) is necessary to meet the indoor lighting requirement. Meanwhile, as a thermochromic smart window, better energy saving performance can be achieved with higher *ΔT*_*so*l_ (e.g. > 10%). Based on this criterion, VO_2_ with 50 nm thickness is selected in the optimized structure since the relatively moderate *T*_*lum*_ and *∆T*_*sol*_ larger than 55% and 11% are achieved, respectively.

### The effect of TiO_2_ layer thickness on the *T*_lum_ and ∆*T*_sol_

**Figure 4:**
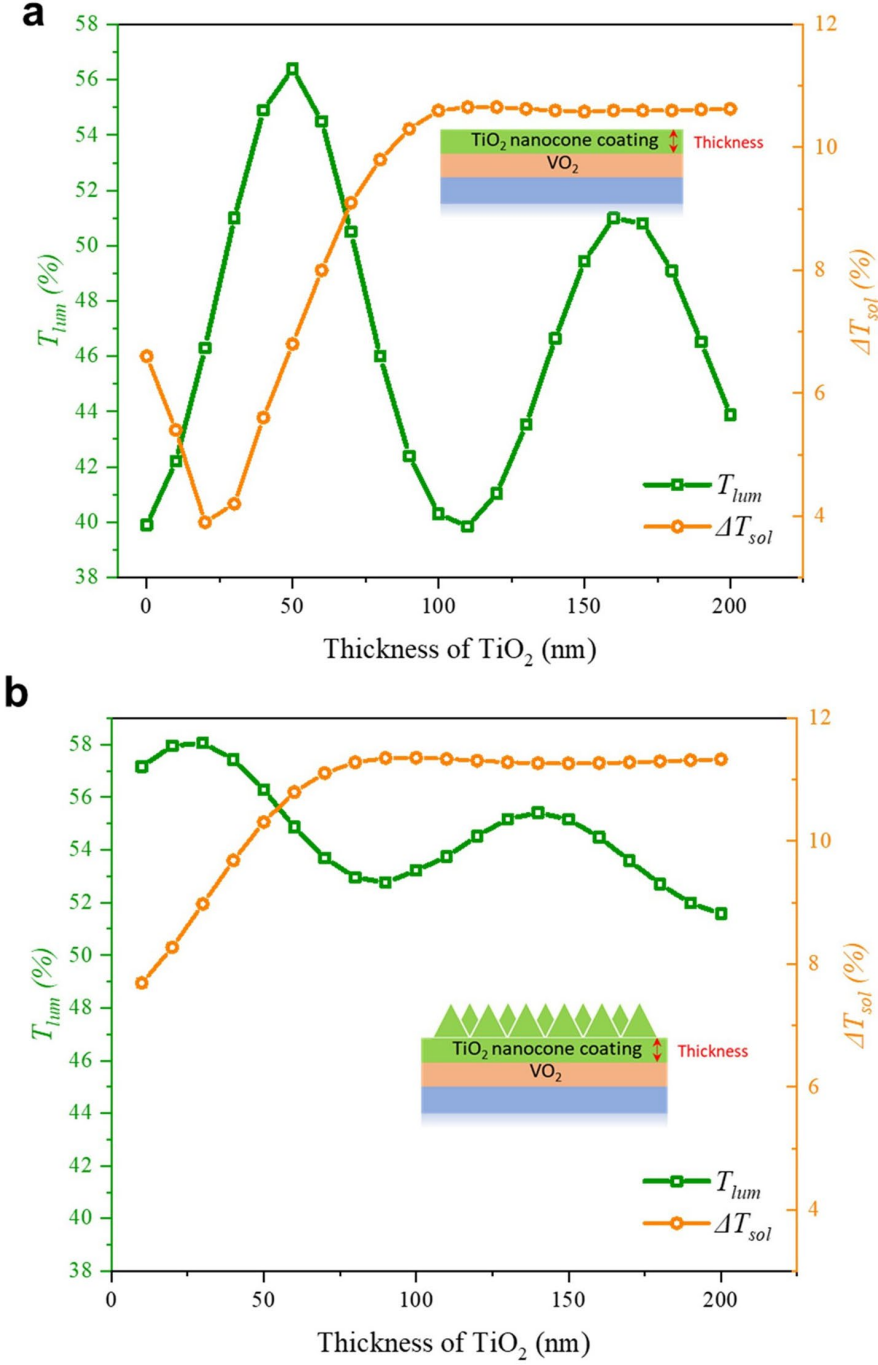
Simulated *T*_*lum*_ and *ΔT*_*sol*_ of different thicknesses of TiO_2_ (**a**) without and (**b**) with nano-cone.

A suitable thickness of the planar TiO_2_ can improve the antireflection performance. Figure 4a summarizes the simulation results of the TiO_2_ layer added on the planar VO_2_ (thickness of VO_2_ is 50 nm) with different thicknesses (10–200 nm). The *T*_*lum*_ increases from 42.2% at 10 nm TiO_2_ to the peak of 56.4% at 50 nm TiO_2_ but drops as the thickness reached 100 nm_._ After that, the *T*_*lum*_ grows again reaching 51.0% with a thickness of 160 nm. The simulation results verify the theory of the antireflection layer-thickness calculation based on Eq. (1). The optical thickness of VO_2_ is 139.5 nm at 550 nm (*d*_*VO2*_ = 50 nm, *n*_*VO2*_ = 2.79). To form the quarter-quarter-waved structure, the theoretical thickness of TiO_2_ (*n*_*TiO2*_ = 2.44 at 550 nm) is around 55 nm. Similarly, for the quarter-three-quarter waved structure, the thickness of TiO_2_ is around 165 nm. The simulation results of TiO_2_ thicknesses at 50 and 160 nm lead to peaks of *T*_*lum*_. The simulation values of TiO_2_ thickness are close to the theoretical values of TiO_2_ thickness. This also confirms the accuracy of the FDTD model. However, the transmittance peaks shift to the left a little after depositing the nano-cone (pitch: 100 nm and height: 250 nm) (Fig. 4b), so the required TiO_2_ thickness of the optimized structure is smaller after adding the nano-cone.

**Figure 5:**
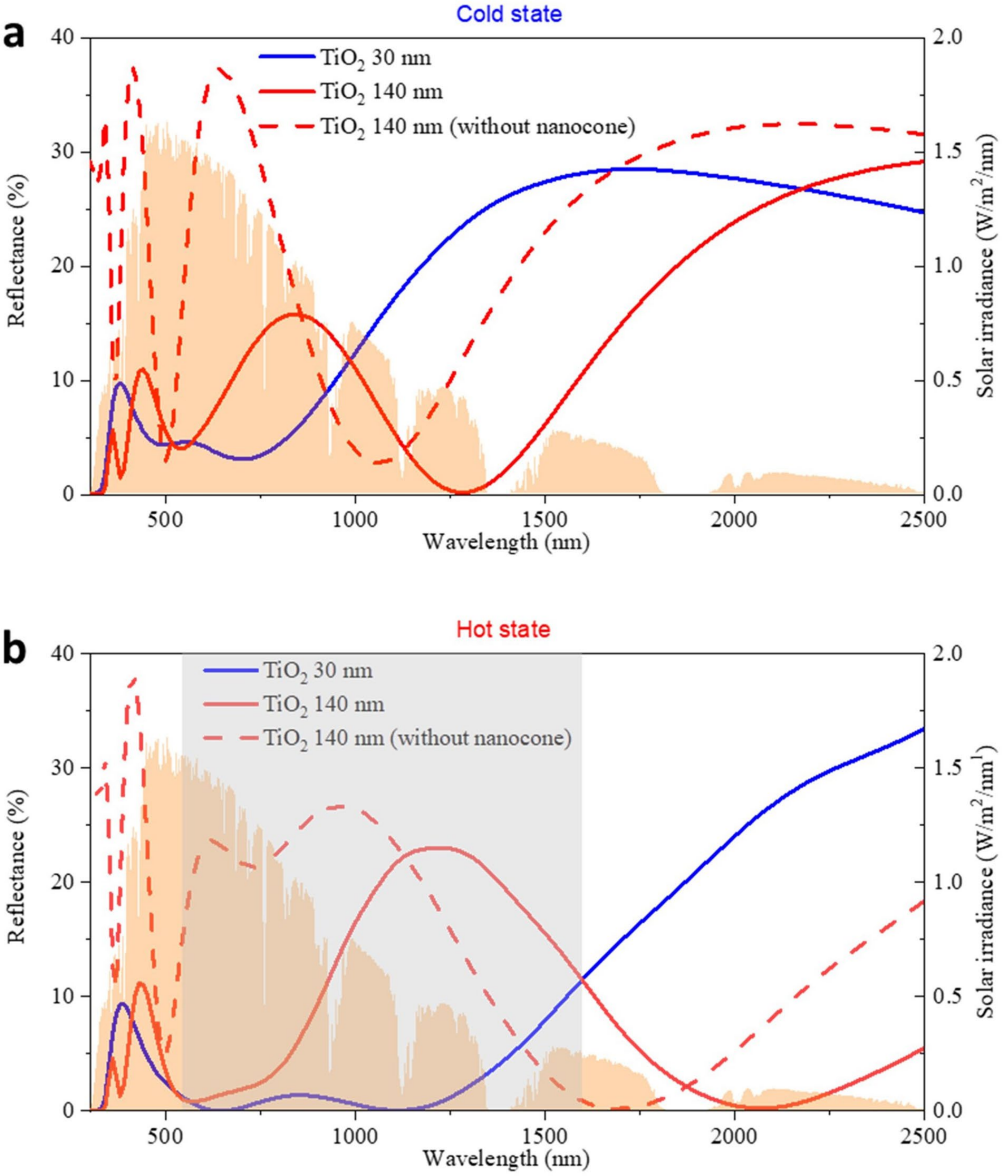
The reflection spectrum of VO_2_ adding 30 nm and 140 nm of TiO_2_ (**a**) at the cold state and (**b**) at the hot state. (Orange filled spectrum is the solar intensity spectrum). The solid line represents the structure with nano-cone (pitch: 100 nm, height: 250 nm). The dash line represents the structure without nano-cone.

The simulation results in Fig. 4b reveal that 30 nm and 140 nm TiO_2_ after adding the nano-cone leads to the two relatively high transmittance vertexes with *T*_*lum*_ at 58.1% and 55.4%, respectively. However, these two thicknesses show different improvements of *∆T*_*sol*_. While *∆T*_*sol*_ can be improved from 6.6% to 9.0% (i.e. improved by 36.7%) after depositing 30 nm of TiO_2_ compared with the pure planar VO_2_, it can reach 11.3% (i.e. improved by 71.6%) at 140 nm of TiO_2_. This difference can be explained by the stronger reflectance of near infrared light after adding 140 nm of TiO_2_ in the hot state. The red and blue solid lines in Fig. 5 illustrate the reflection spectra of VO_2_ after adding 30 nm and 140 nm of TiO_2_ respectively. It is observed that regardless of the cold or hot state, after adding 30 nm and 140 nm of TiO_2_, the relative low reflectance appears in the visible light region. This is the key reason why *T*_*lum*_ can be significantly improved due to the good antireflection performance in the visible light region. But the reflection spectrums are quite different in the NIR region (780 nm to 2,500 nm). Although the spectrum shapes of adding 30 nm and 140 nm TiO_2_ are different in Fig. 5a, the reflectance of 30 nm and 140 nm TiO_2_ are almost identical in the cold state, which induces a small difference of *T*_*sol,cold*_ between the 30 nm and 140 nm TiO_2_ layers (56.5% vs 55.7%). However, the reflectance of *T*_*sol,hot*_ by adding 140 nm of TiO_2_ is 44.5% which is lower than adding 30 nm TiO_2_ whose *T*_*sol,hot*_ is 47.6%. This is because the huge reflectance from 550 nm to 1,600 nm (shaded area in Fig. 5b), where there exists strong solar irradiance, leads to the decrease of *T*_*sol,hot*_ after the addition of 140 nm of TiO_2_. The smaller *T*_*sol,hot*_ enhances the *∆T*_*sol*_ on the condition that *T*_*sol,cold*_ is almost the same when 30 nm and 140 nm of TiO_2_ is added. The nano-cone can also help reduce the reflection in a broadband wavelength range: comparing the red solid line and red dashed line in Fig. 5, the reflection is significantly reduced over the whole wavelength from 300–2,500 nm. For example, in Fig. 5a, there are two reflection peaks from 370 to 500 nm and 500 nm to 1,060 nm (red dashed line). However, the reflectance in these two broad ranges is significantly suppressed after adding the nano-cone. This unique feature of nano-cone is quite competitive compared with the planar antireflection layer which can only reduce reflection at specific wavelengths. Since the addition of 140 nm TiO_2_ leads to a larger *∆T*_*sol*_ and comparable *T*_*lum*_ relative to 30 nm TiO_2_, 140 nm TiO_2_ is chosen for the optimized structure.

### The influence of TiO_2_ nano-cones with different dimensions of pitch and height on the *T*_lum_ and ∆*T*_sol_

Researchers have established a comprehensive optical theory about the nano-cone antireflection structure^29,32,34,49,50^. Based on the equation introduced in these theories, to avoid the structures being resolved by the incident light, the pitch (*P*) should satisfy the following Eq.^51^:

10 $$ P \le \frac{\lambda }{{n_{TiO2} + n_{air} \sin \left( \theta \right)}}, $$

where *n*_*TiO2*_ and *n*_*air*_ are the refractive index of TiO_2_ and air, respectively; *θ* is the angle of the incident light, that was set to 0 (normal incident light) in the following simulations; *λ* is the wavelength of the incident light. The main function of depositing the nano-cone is to increase luminance transmittance, therefore, 780 nm became the targeted wavelength for the following antireflection design, and the pitch of the nano-cones should be smaller than 226 nm according to Eq. (10). The height of the nano-cones can also strongly influence the optical performance. It is found that reflectance can be dramatically reduced with higher nano-cones^29,50^.

**Figure 6:**
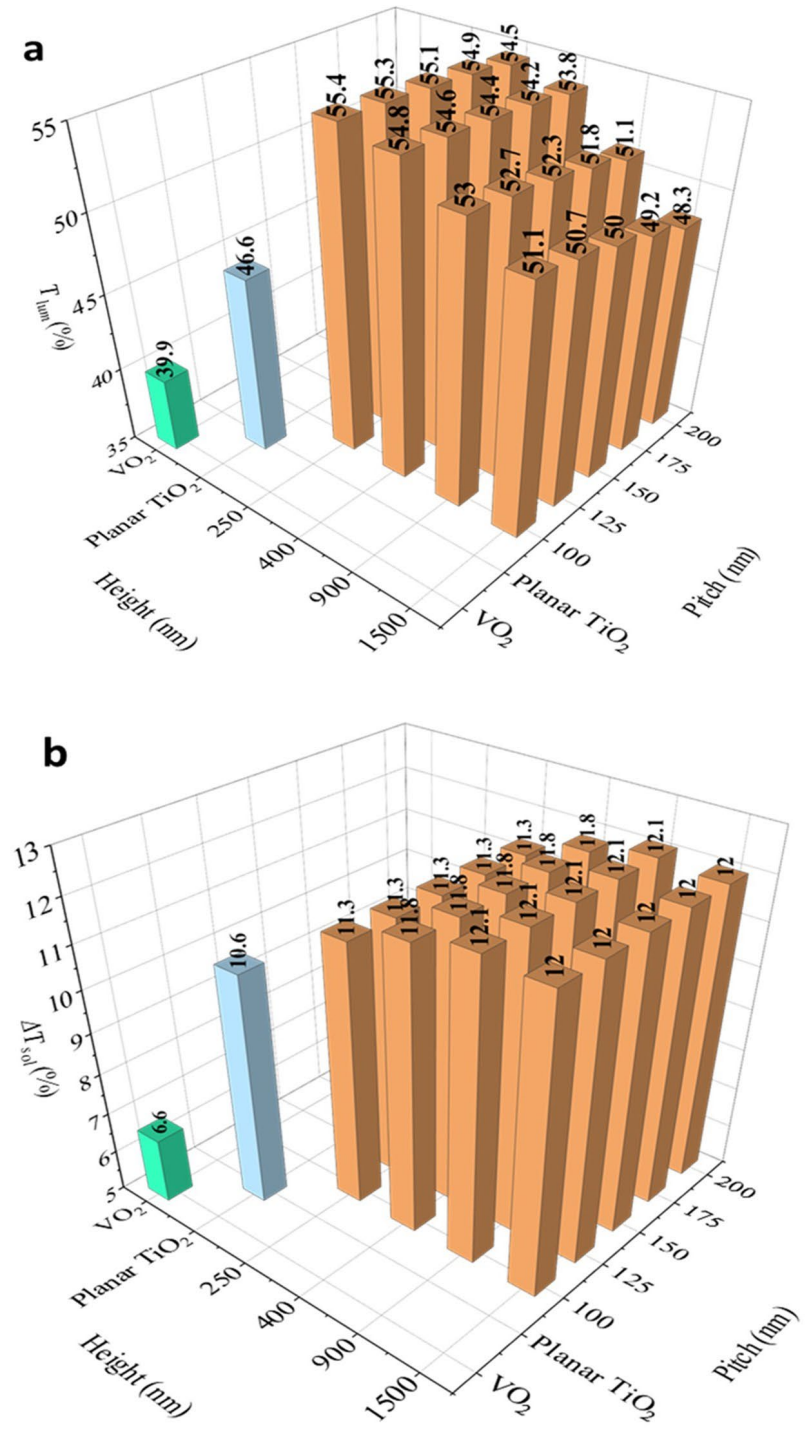
(**a**) Simulated *T*_*lum*_ and (**b**) simulated *∆T*_*sol*_ at different pitches and heights of nano-cones.

Based on the guidelines, we investigate the pitch of 100 nm, 125 nm, 150 nm, 175 nm, 200 nm, and height of 250 nm, 400 nm, 900 nm and 1,500 nm. The simulation results are shown in Fig. 6. In Fig. 6a, the *T*_*lum*_ of 50 nm VO_2_ (structure 1) is 39.9% while the *T*_*lum*_ adding 140 nm TiO_2_ (structure 2) is 46.6%. It is found that when the pitch of the nano-cones is smaller than 200 nm, the highest transmittance can be achieved at 55.4%. Figure 6a illustrates the change of *T*_*lum*_ with a pitch smaller than 200 nm; the smaller pitch and lower height can lead to higher transmittance. However, nano-cones with larger pitch and height can lead to lower transmittance because of the strong absorption (*A*_*lum*_) for the larger height (Supplementary Fig. S6a). Our simulation results also show that the *R*_*lum*_ is reduced to less than 4.1% after adding the nano-cones (Supplementary Fig. S6b), proving that the nano-cone structures are effective as antireflection layers. However, the absorbance is enhanced as the cone size increases. Regarding *∆T*_*sol*_, the simulation results (Fig. 6b) do not reveal a significant change in the solar modulation ability when the pitch size varies. The *∆T*_*sol*_ varies from 11.3% with a nano-cone height of 250 nm to 12.1% with a height of 900 nm.

### Optical performance of optimized structure

Based on the simulation results, the suitable pitch size should be smaller than 200 nm, as a smaller pitch leads to higher transmittance. Besides, the transmittance decreases as the height increases. However, the higher cone can induce a slight positive effect (~ 0.7% absolute) on the solar modulation ability. For the feasibility of practical fabrication, the ratio of height to pitch has to be small. Hence, it is suggested the optimized nano-cone dimension range is 200 nm ≥ pitch ≥ 100 nm, and 400 nm ≥ height ≥ 250 nm. It is expected that a high transparency *T*_*lum*_ > 55% with a moderately high *ΔT*_*sol*_ > 11% could be obtained with the optimized structure.

**Figure 7:**
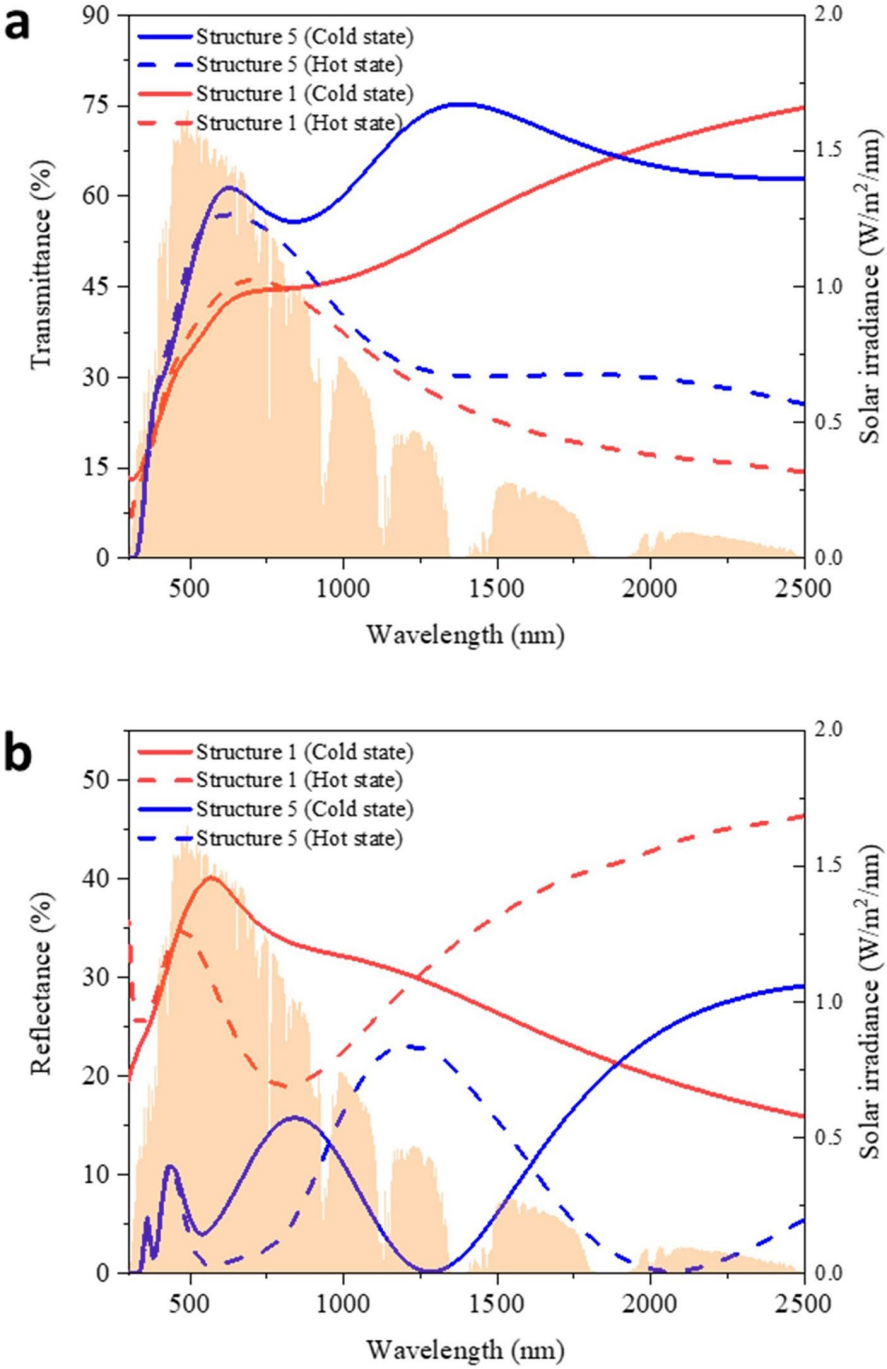
(**a**) Transmittance spectrum and (**b**) reflection spectrum of VO_2_ smart windows with nano-cones (structure 5) and without nano-cones (structure 1) (Nano-cone dimension: Pitch: 100 nm, Height: 250 nm; Orange filled spectrum is the solar intensity spectrum).

Figure 7a clearly demonstrates the transmittance in the visible light region is significantly enhanced by the nano-cones (structure 5-pitch: 100 nm, height: 250 nm, TiO_2_: 140 nm, VO_2_: 50 nm) compared with the planar VO_2_ windows (structure 1- VO_2_: 50 nm). The optimized structure exhibits *T*_*lum*_ of 55.4% with *ΔT*_*sol*_ of 11.3%, an improvement of about 39% and 72%, respectively, compared with structure 1. A comparison of the reflectance spectra is shown in Fig. 7b. Based on these reflectance spectra, it is shown that *R*_*lum*_ dropped from 39.2 to 5.2% in the cold state and from 30.2 to 1.5% in the hot state after adding the nano-cones, which contributes to the improvement of transmittance. For the VO_2_-based smart windows, the solar modulation is mainly due to the regulation in the NIR region. The calculation results based on Fig. 7a show that *T*_*IR*_ in the cold state increases from 50.8% to 62.3%, while *T*_*IR*_ in the hot state does not change as significantly as the cold state after adding the nano-cones, only from 33.3 to 37.9%. The reason is that the enhancement of the transmittance in the cold state is mainly at the 1,000–2,000 nm region, where the solar irradiance is strong, but the transmittance in the hot state is enhanced at the relatively long wavelength region (i.e. 1,500–2,500 nm) where the solar irradiance is low. The stronger transmittance of NIR in the cold state enhances the *T*_*sol,cold*_, benefiting the *ΔT*_*sol*_*.*

Moreover, to prove the functional purpose of using TiO_2_ nano-cone deposited on the planar TiO_2_, one more simulation is conducted. Three different structures, including structure 5 (here, it is re-named as Structure A), Structure B that is TiO_2_ nano-cone directly deposited on the VO_2_ layer, and Structure C that is VO_2_ nano-cone deposited on top of the 50 nm VO_2_ thin film are investigated. The results are illustrated in Supplementary Fig. S7, and found that the optimized Structure B shows a slightly higher *T*_*lum*_ than that of Structure A (i.e. 56.6% vs 55.4%). However, *ΔT*_*sol*_ is extremely low (i.e. 7.6%) compared to Structure A (i.e. 11.3%). The optimized Structure C demonstrates a relatively high *ΔT*_*sol*_ (i.e. 16.1%), and this can be explained by the VO_2_ being thicker after depositing the VO_2_ nano-cones. However, the *T*_*lum*_ of Structure C is only 39.4% which is even lower than that of the planar VO_2_ structure (i.e. 39.9%). The high absorption of VO_2_ severely weakens the function of the nano-cone. All in all, these comparisons indicate that the TiO_2_ nano-cones deposited on the planar TiO_2_ layer can achieve relatively high performance in both *T*_*lum*,_ and *∆T*_*sol*_ . In addition, thanks to the self-cleaning property^52,53^ and relatively mature imprinting technique of TiO_2_^37–40,54^, the usage of TiO_2_ on thermochromic smart window applications definitely provides the versatility and maneuverability in future studies.

**Figure 8:**
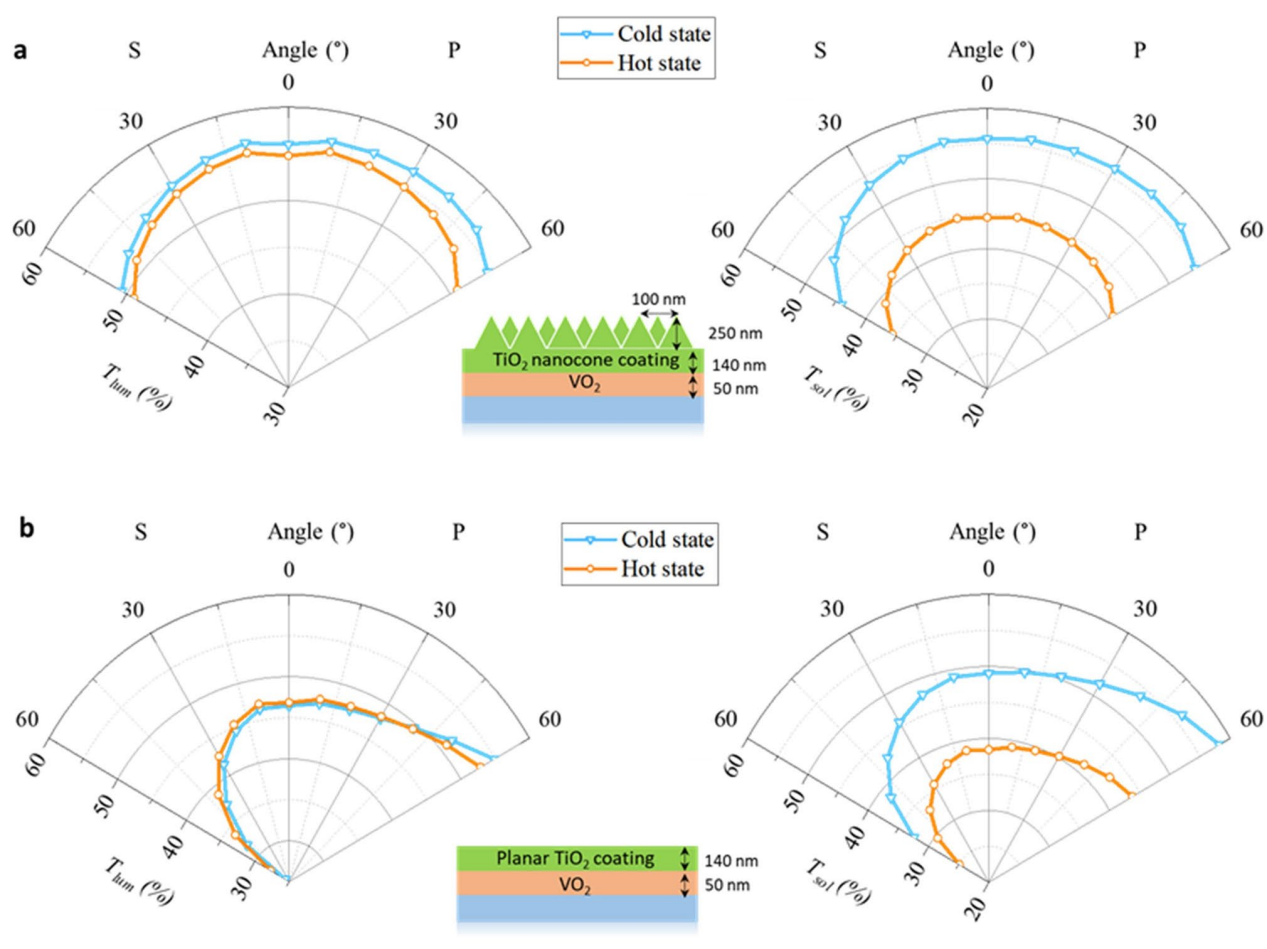
The simulation results of *T*_*lum*_ and *T*_*sol*_ of the smart window (**a**) with nano-cone and (**b**) without nano-cone, under different angles and polarization state. *P* p-polarized light, *S* s-polarized light.

It should be noted that natural light shows a random degree of polarization, including p- and s-polarizations, which have the electric field perpendicular and parallel to the incidence plane, respectively. Good optical performance for both p- and s-polarizations is required for the windows. To compare the *T*_*lum*_ and *ΔT*_*sol*_ of the planar TiO_2_ antireflection layer and nano-cone structure under different polarization state, the simulations of incidence angles up to 60° under p- and s-polarized light are conducted. The optimized dimension of the selected nano-cone structure is a pitch of 100 nm with height of 250 nm (structure 5). The simulation results are compared with the planar 50 nm VO_2_ after adding 140 nm of TiO_2_ (structure 2). Figure 8 shows that the nano-cone structure can function well over a wide range of angles and different polarization light compared with the planar structure. The planar TiO_2_ antireflection layer shows a strong angular and polarization dependence, especially for the *s*-polarized light. The luminous transmittance drops from 47 to 27% from normal incidence to 60° incidence with the decrease of *ΔT*_*sol*_ from 10.6 to 7.6% under *s*-polarized light. Nevertheless, the *T*_*lum*_ and *ΔT*_*sol*_ remain higher than 49.8% and 8.4% respectively for both p- and s-polarized light from 0° to 60° after adding the nano-cones. The simulation results confirm that the nano-cone structure is less sensitive to the direction and polarization of the optical source. This property can ensure the nano-cone structure exhibits high *T*_*lum*_ and *ΔT*_*sol*_ under different incident angles of natural light, which is more practical for the real application compared with planar structures.

### Comparisons with other work

Table 1 compares previous reports with this work in improving VO_2_ thermochromic smart window performance (*T*_*lum*_ and *ΔT*_*sol*_) using different strategies. It should be noted that only pure VO_2_ and only one layer of TiO_2_ is deposited as the planar antireflection layer in this study to show the optical enhancement of TiO_2_ nano-cones. It is believed that with a multi-layer structure (e.g. TiO_2_/VO_2_/TiO_2_/Nano-cone) integrated with modified VO_2_ (e.g. Chemical doping VO_2_ or VO_2_ nanoparticles), the *T*_*lum*_ and *ΔT*_*sol*_ can be further enhanced. Most importantly, angular and polarization independence of this structure cannot be easily achieved by other strategies. This unique property can ensure that the VO_2_ smart window retains the high optical performance under natural sunlight.

**Table 1:** Summary of enhanced strategies, material, structure design and optical properties of VO_2_ thermochromic smart windows.

Enhanced strategies	Material/structure	Category	*T*_*lum*_ (%)	*ΔT*_*sol*_ (%)	Refs
Embedded VO_2_ nanoparticles	TiO_2_	Experiment	61.2	14.6	^8^
	PDMS	Experiment	85.0	*/*	^9^
	Doped W	Experiment	56.0	12.7	^10^
	Si-Al gel	Experiment	59.1	12.0	^11^
VO_2_ morphology modification	Nano-grid	Simulation	76.5	14.0	^14^
		Experiment	67.0	8.8	^15^
	Porosity (polymer-assisted deposition method)	Experiment	50.0	14.7	^12^
	Porosity (freeze drying method)	Experiment	41.6	14.1	^13^
Depositing antireflection layer	Planar structure-TiO_2_/VO_2_	Experiment	49.0	7.0	^21^
	Planar structure-TiO_2_/VO_2_/TiO_2_	Experiment	57.6	2.9	^22^
	Planar structure-TiO_2_/VO_2_/TiO_2_/VO_2_/TiO_2_	Experiment	43.7	12.1	^24^
	Planar structure-TiO_2_/VO_2_/SiO_2_	Experiment	61.5	6.9	^42^
	Bioinspired moth eye-nipple arrays	Simulation	70.3	23.1	^23^
		Experiment	44.5	7.1	^5^
	Bioinspired moth eye-nano cones	Simulation	55.4	11.3	(This work)

## Conclusion

Inspired by moth eyes, this study proposes a novel TiO_2_ nano-cone structure antireflection layer to achieve high *T*_*lum*_ and *ΔT*_*sol*_ for VO_2_ thermochromic smart window applications. FDTD simulation is conducted to achieve the design rules and identify the optimal dimensions. The results show that high transparency (> 55%) with moderately high *ΔT*_*sol*_ (> 11%) can be achieved by the optimized nano-cone dimensions with 100 nm pitch and 250 nm height coated on 140 nm of TiO_2_/50 nm VO_2_. The improvements of *T*_*lum*_ and *ΔT*_*sol*_ are about 39% and 72%, respectively, compared to the single layer VO_2_ coated thermochromic smart window. The enhancement of the optical performance provides a brighter and more energy-efficient indoor environment. Furthermore, the wide-angle simulations demonstrate that the nano-cone structure exhibits better angular and polarization independence than the planar TiO_2_ antireflection layer for VO_2_ smart windows. This property makes VO_2_ smart windows more practical under natural sunlight. The simulation of this work demonstrates that the TiO_2_ nano-cone antireflection layer approach is unique for providing high *T*_*lum*_ and *ΔT*_*sol*_ under wide angle space and different polarization directions. It should be noted that the TiO_2_ nano-cone can be fabricated by nanoimprint lithography of sol–gel derived TiO_2_ layers^54–56^. The imprinting process is industrial friendly for roll-to-roll or large area fabrication, which makes the nanoimprint lithography process lower cost^54,57^. The nano-cone structure can also be easily integrated with modified VO_2_ or other thermochromic materials to develop more efficient thermochromic smart windows. This study opens a new way to develop high performance thermochromic smart windows. The proposed TiO_2_ nano-cone coating is very promising because of its high transmittance in broadband wavelength, high solar modulation, and polarization-insensitivity. It facilitates applications of VO_2_ thermochromic smart windows as a sustainable envelope system for energy-efficient buildings.

## Supplementary information

Supplementary information

## Data Availability

The datasets generated during and/or analyzed during the current study are available from the corresponding author on reasonable request.
