# Supplementary material for: Bio-inspired TiO2 nano-cone antireflection layer for the optical performance improvement of VO2 thermochromic smart windows
Source: Sci Rep. 2020 Jul 9;10:11376. doi: 10.1038/s41598-020-68411-6 (PMC7347835; doi:10.1038/s41598-020-68411-6)
Supplement: Supplementary file 1 — Supplementary information [file 41598_2020_68411_MOESM1_ESM.docx]

Supplementary information for

Bio-inspired TiO_2_ Nano-cone Antireflection Layer for the Optical Performance Improvement of VO_2_ Thermochromic Smart Windows

Sai Liu^1^, Chi Yan Tso^1*^, Hau Him Lee^1^, Yi Zhang^2^, Kin Man Yu^3^, & Christopher Y.H. Chao^2^

^1^School of Energy and Environment, City University of Hong Kong, Tat Chee Avenue, Kowloon, Hong Kong, China

^2^Department of Mechanical Engineering, The University of Hong Kong, Hong Kong, China

^3^Department of Physics, City University of Hong Kong, Tat Chee Avenue, Kowloon, Hong Kong, China

* Corresponding Author Tel.: +852 3442 4623; E-mail Address: [chiytso@cityu.edu.hk](mailto:chiytso@cityu.edu.hk)


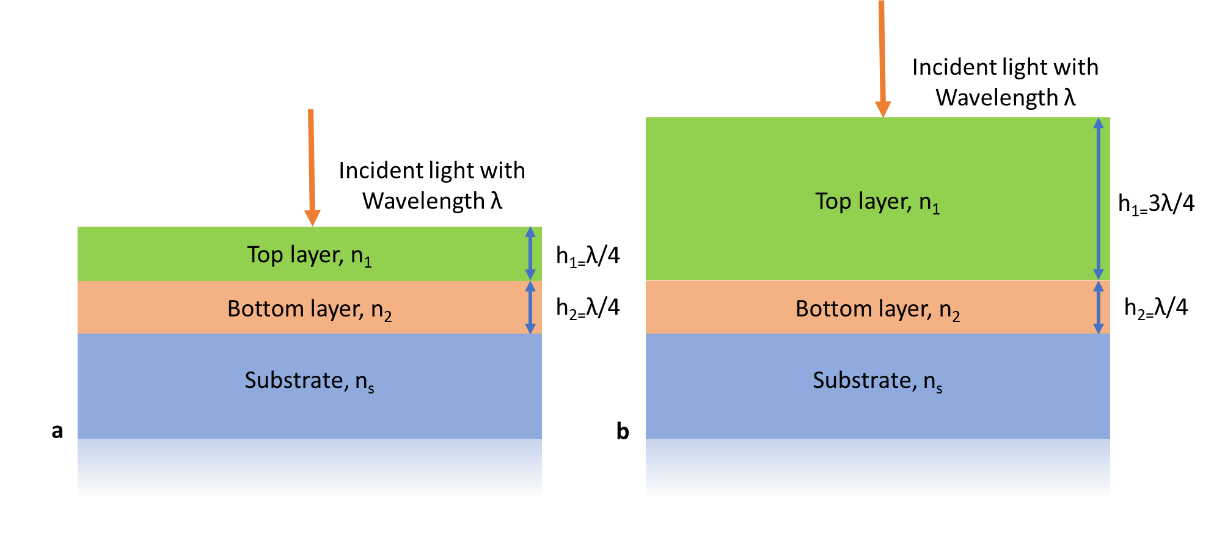


**Supplementary Figure S1**. Two commonly used antireflective structures: (a) quarter-quarter-waved antireflective structure and (b) quarter-three quarter-waved antireflective structure. In this study, VO_2_ film is the bottom layer, TiO_2_ is the top layer, and quartz is the substrate.

**Supplementary Figure S2**. The spectrum of the photopic luminous efficiency of the human eye (green region) and the AM1.5 solar irradiance (orange region).


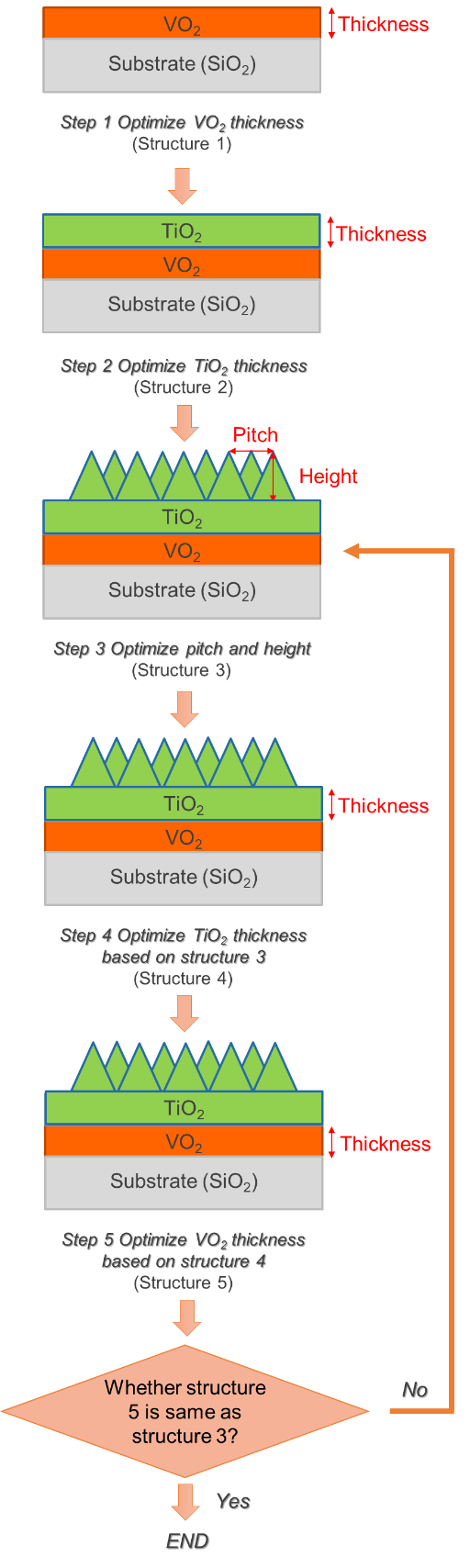


**Supplementary Figure S3**. Optimization steps (the words in red are the optimized parameter in each step), the optimized structures with high *T_lum_* and *ΔT_sol_* in each step were named as structure 1, structure 2, structure 3, structure 4 and structure 5.


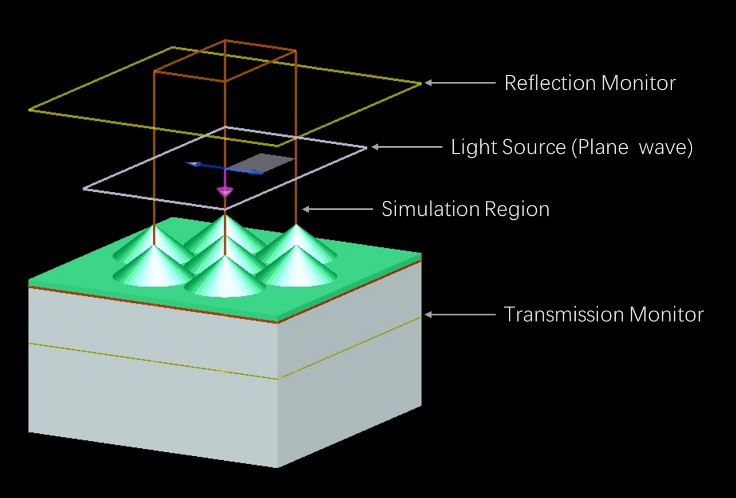


**Supplementary Figure S4**. Graphical illustration of the FDTD simulation model.

**Supplementary Figure S5**. *T_lum_* and *ΔT_sol_* comparisons between the simulation model in this study and Ref. 1 of a single layer VO_2_ smart window.

**
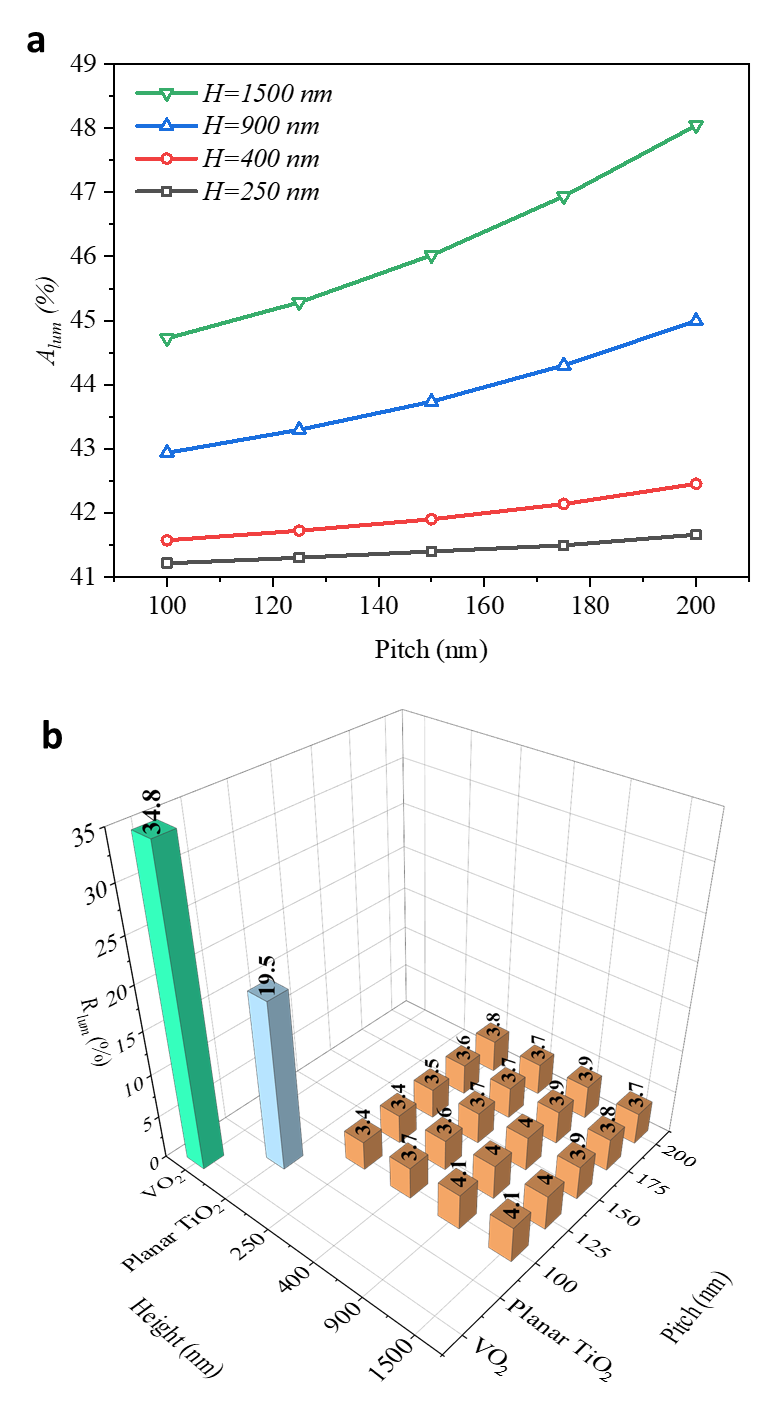
**

**Supplementary Figure S6**. (a) Simulated *A_lum_* and (b) simulated *R_lum_* at different pitches and heights (*H*) of nano-cones.


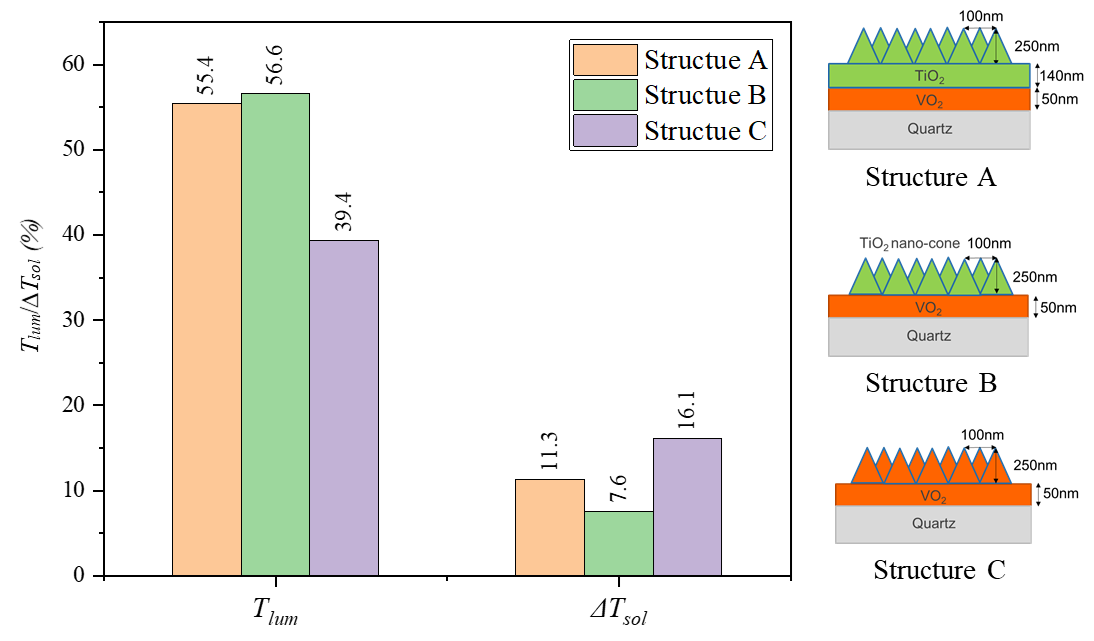


**Supplementary Figure S7**. *T_lum_* and *ΔT_sol_* comparisons among different structures.

**Supplementary Note S1:** The optimization was conducted by the following steps sequentially, *T_lum_* as well as *ΔT_sol_* are calculated to select the most suitable case for the following steps. In order to avoid confusion and for convenience, the optimized structures in each step were named as structure 1, structure 2, structure 3, structure 4 and structure 5, respectively, as shown in Supplementary Fig. S3: Step 1, the thickness of the VO_2_ deposited on the quartz _­_substrate was determined. Next, Step 2, the TiO_2_ layer was added and the thickness was optimized. Step 3, the nano-cones with different height and pitch were coated to examine the best combination. However, step 3 may influence the interface reflection which is crucial for the results of steps 1 and 2. To ensure the optimization can reflect the interaction of all the layers, steps 4 and 5 are conducted accordingly. In step 4, only the thickness of TiO_2_ is optimized, while the thickness of VO­_2_, the pitch and height of the nano-cone remain as structure 3. Similarly, in step 5, only the thickness of VO_2_ is optimized whereas the thickness of TiO_2_, the pitch and height of the nano-cone are the same as structure 4. The optimization process is cycled until the dimension of each parameter in structure 5 is identical to structure 3.

**Supplementary Table S1**. *T_lum_* and *ΔT_sol_* comparisons between the simulation model in this study and Ref. 2 of glass/TiO_2_/VO_2_/TiO_2_/VO_2_/TiO_2_ five-layer structure

|  | *T_lum_* (%) | | *T_sol_* (%) | |
| --- | --- | --- | --- | --- |
|  | Cold state | Hot state | Cold state | Hot state |
| Simulation results based on the model of this study | 35 | 29 | 43 | 29 |
| Experiment results of Ref. 2 | 39 | 31 | 42 | 28 |

**References**

1. Taylor, A. *et al.* A bioinspired solution for spectrally selective thermochromic VO_2_ coated intelligent glazing. *Opt. Express* **21**, A750 (2013).
2. Mlyuka, N. R., Niklasson, G. A. & Granqvist, C. G. Thermochromic VO_2_-based multilayer films with enhanced luminous transmittance and solar modulation. *Phys. Status Solidi Appl. Mater. Sci.* **206**, 2155–2160 (2009).
